# Development of a Unique Student Pharmacist Internship in a Primary Care Provider System

**DOI:** 10.3390/pharmacy7020036

**Published:** 2019-04-13

**Authors:** Norman E. Fenn, Natalie R. Gadbois, Gwen J. Seamon, Shannon L. Castek, Kimberly S. Plake

**Affiliations:** 1 Fisch College of Pharmacy, The University of Texas at Tyler, Tyler, TX 75799, USA; nfenn@uttyler.edu; 2 College of Pharmacy, Purdue University, West Lafayette, IN 47907, USA; ngadbois@purdue.edu (N.R.G.); gjseamon@gmail.com (G.J.S.); scastek@gmail.com (S.L.C.)

**Keywords:** pharmacy intern, student pharmacist, primary care, interprofessional, federally qualified health center

## Abstract

Purpose: To describe a unique pharmacy intern program in a group of federally qualified health center (FQHC) outpatient primary care provider clinics. Summary: A pharmacy intern program was created at the North Central Nursing Clinics in Indiana, a group of four FQHC outpatient primary care provider facilities. Intern-performed tasks included: Prior authorization (PA) requests, medication assistance program (MAP) applications, sample procurement and inventory, and contraceptive devices for implantation inventory management. Interns interacted with clinic administration, nurse practitioners, and medical staff to complete their assigned responsibilities. Over a one-year period, the interns completed documentation on more than 2000 charts during a combined 12 h a week. Interns identified the interprofessional interactions as the most beneficial experience, while providers acknowledged no difference in the processing of paperwork during the transition of duties from pharmacy fellow to intern. Conclusion: This unique pharmacy intern program was successfully created and implemented in a primary care provider office, resulting in learning opportunities for pharmacy interns, as well as operational efficiencies to fellows, providers, and the organization.

## 1. Introduction

Pharmacy interns have a variety of different roles and responsibilities and typically work in community and hospital pharmacy environments [1,2,3,4,5,6]. Student pharmacists, serving as interns, contribute to their educational process and professional development as they gain practical experiences related to the pharmacy curriculum. While there is evidence of student pharmacist involvement in direct patient care activities through student-run free clinics, these students operate on a volunteer basis or are participating as part of a required pharmacy practice experience, compared to intern positions that are paid [7,8,9,10]. Currently, there are no descriptions of the role of pharmacy interns in the literature beyond those in hospital or community pharmacy settings. In this manuscript, a unique pharmacy intern program in a rural outpatient primary care facility is described.

The North Central Nursing Clinics (NCNC) in Indiana consist of four primary care provider offices in rural communities. Affiliated with the Purdue University School of Nursing and College of Pharmacy, the clinics are led by nurse practitioners whose specialties include pediatrics, family medicine, and women’s health. Other integral members of the healthcare team include support staff (e.g., front office personnel, billing, insurance navigators), social workers, medical assistants, and nurses. The clinics are federally qualified health centers (FQHC), and all four sites are considered Level 2 Patient-Centered Medical Homes. As part of the FQHC requirements, no patient is denied care based on their ability to pay for healthcare services. The patient population served at each of the clinics varies, with 42–45% of patients enrolled in Medicaid and/or Medicare and 22–23% of patients being uninsured. Roughly 10% of the clinics’ patients are native Spanish speakers, requiring translational and interpretive services.

Pharmacy fellows are licensed pharmacists who serve as the sole provider of clinical pharmacy services at the clinics as part of the Purdue University College of Pharmacy Academia and Ambulatory Care Fellowship program. Fellows spend approximately 50% of their time in clinic and perform multiple roles in the clinics, including: conducting patient visits for chronic diseases under collaborative practice agreements (CPAs), precepting student pharmacists on advanced pharmacy practice experiences (APPEs), and providing cost-effective medication recommendations. Fellows are also responsible for completing prior authorizations (PAs), assisting patients in obtaining medication through medication assistance programs (MAPs), and drug inventory management. Completion of a postgraduate year one residency is a prerequisite for the fellowship program, as the fellows are required to operate independently at these clinics. Two of the clinics serve as the fellows’ primary practice sites, with one fellow based at each clinic. In addition, each fellow provides remote pharmacy services for the clinics not staffed by the fellowship program.

## 2. Pharmacy Internship Program Description and Review

In August 2016, pharmacy services expanded, which resulted in increased direct patient care activities and the need for assistance with administrative tasks. As a response to this need, the pharmacy internship program was created. Two interns were hired for a total of 12 h per week. Described duties included completing PAs, coordinating MAP applications, and tracking contraceptive device prescription processing for each of the four clinics, as well as managing inventory of medication samples at the main clinic. It was decided by all involved parties that students recruited for the intern positions would have completed at least their first year of the professional program. Both interns are supervised by the on-site fellow and communicate any issues or questions to them. The interns each work primarily out of one clinic site location, but assist with administrative responsibilities for patients from all of the clinics. All documentation by the interns is submitted to both the responsible provider and the fellow for review. Interns also perform follow-up phone calls with the patient and the pharmacy as necessary to ensure timely processing of medications and expedite patient care. When processing PAs, if an adjustment can be made due to a formulary requirement or therapeutically equivalent medication, the fellow is authorized to change the medication order, either through a CPA or with verbal authorization from the provider.

From October 2017 to October 2018, pharmacy interns completed in excess of 2000 documented tasks (Table 1). Interns initially worked a combined 12 h a week at the clinic. For quality assessment, interns were instructed to document their daily workload processes, recording the number of PAs, MAPs, contraceptive devices, inventory and other assorted tasks not covered under these categories. As a result of significant work volume, intern hours were expanded to a combined 16 hours a week in May 2018. The documented tasks may be slightly underestimated due to some brief gaps in data recording. In addition to the routine activities outlined previously, examples of some unique intern tasks include working with a pharmaceutical company to complete an adverse event report in response to a vaccine and clarifying dosing discrepancies on prescriptions with community pharmacies.

## 3. Benefits of the Pharmacy Internship Program to Pharmacy Interns

The Fink Model of Significant Learning (FMSL) was applied in the development of the internship. In this model, Fink outlined six main categories of learning that create significant learning experiences for students. These six categories are: (1) foundational knowledge, (2) application, (3) integration, (4) human dimension, (5) caring, and (6) learning how to learn. Interns experienced each of these categories as a part of the internship program. The interns are utilizing their foundational knowledge of medication use from the didactic curriculum and integrating specific patient needs as part of the PA and MAP processes. Through their interaction with providers and patients, they are learning about the human dimension of patient care, as well as caring and advocating for others. Interns regularly inquire, or learn how to learn, to resolve drug-related problems and issues related to medication access [11].

Pharmacy interns participated in semi-structured interviews upon completion of their term. The pharmacy interns describe this position as rewarding for several reasons. Primarily, it offers an opportunity to work with vulnerable and underserved populations, a unique experience not always provided through traditional internships (FMSL categories: caring and human dimension). The diverse patient population requires multi-faceted approaches to obtaining medications that are often expensive. Interns regularly review pharmaceutical companies’ MAPs, community pharmacy discount medication programs, discount medication cards, and websites (FMSL categories: Learning how to learn and application). Interns are also offered opportunities to shadow providers on their own unpaid time, if desired, to potentially gain a greater understanding of interprofessional relationships and comprehensive patient care that occurs in an ambulatory care setting. Additionally, interns participate in scholarly opportunities related to their work, resulting in two poster presentations and two manuscript preparation opportunities to date (FMSL categories: Learning how to learn, integration, and application). Finally, interns educate other student pharmacists who are completing curricular requirements, such as a rotation or service-learning activity about the intern responsibilities at the clinic (FMSL categories: Integration and human dimension).

Prescriber management of PAs is often unseen by student pharmacists. Both interns previously completed an introductory pharmacy practice experience (IPPE) in the community setting and observed the PA process in that setting. As processing PAs is the primary function of the intern position, it exposes interns to the challenges faced when working with a variety of insurance companies, while also ensuring patients receive their necessary prescribed medications. In processing PAs, interns critically review patient profiles and determine the patient-specific factors that are relevant in the selection of a medication (FMSL categories: foundational knowledge, application, and integration). Interns further advocate for patients with insurance companies by communicating the provider’s rationale and decision-making process in prescribing the requested medication (FMSL categories: Caring, human dimension, and integration). Interns take part in an essential component of the prescribing process, leading to a better understanding of preferred medications, insurance formularies, and prior authorization approval criteria of prescription medications.

Additionally, the interns valued learning about the financial barriers experienced by some of the NCNC patient population (FMSL category: caring). Patients, especially those who are vulnerable or underserved, often see costs associated with their care as burdensome, and will periodically go without care, leading to poor health outcomes and increased healthcare costs [12]. Interns helped address these barriers by coordinating between providers and patients to complete MAP applications, resulting in increased medication access for patients and potentially improved patient outcomes (FMSL categories: Foundational knowledge, application, and integration). Interns identified barriers which slowed or complicated the MAP process, including the patient’s motivation to provide and complete the required documentation, as well as provider understanding of application requirements. Solutions to these identified barriers were implemented as a result of intern observations, including provider education on the application processes, prefilled applications with provider information already completed, highlighted patient requirements on the applications, and increased availability of applications to providers and patients.

Skills learned by the interns in the pharmacy curriculum were applied and practiced through the internship program. Areas of emphasis include appropriate written communication and thorough documentation, interprofessional collaboration with providers to address insurance and/or patient barriers, and dissemination of key clinical information to insurance companies to justify a medication’s necessity. Additionally, interns apply and integrate many of the tools and skills learned in the didactic portion of their pharmacy curriculum (i.e., foundational knowledge). Their role in the clinic allows them to practice skills prior to becoming pharmacists, when they will be expected to make clinical recommendations based on a variety of patient-specific factors.

## 4. Benefits of Pharmacy Internship Program to the FQHC Clinics

Pharmacist Fellows—Primarily, the pharmacy intern program allowed pharmacist fellows to explore and expand clinical pharmacy services, including an increase in direct patient care activities as a result of decreased time addressing PAs and MAPs. Subsequently, the fellows have implemented CPAs with the providers to manage multiple chronic disease states. The number of patients receiving anticoagulation management services has more than doubled, an event that has been aided by the transfer of administrative responsibilities. Providers are able to refer patients with diabetes to a pharmacist-directed telephone consult program designed to facilitate insulin management. Finally, in supervising the interns, the fellows gain additional management experience that will prove vital in their future careers as clinical faculty and preceptors.

Providers—The pharmacy interns provide on-site coverage for administrative paperwork four days a week, and are able to interact with providers, should the need arise. Interns facilitate MAP applications, obtain provider signatures for PAs, address any inventory issues, and communicate needed changes in a timely and efficient manner. Providers receive education on common prescribing issues with insurance formularies (e.g., prescribing omeprazole instead of Prilosec OTC^®^) and modify their prescribing habits to avoid these issues. Providers and their patients have also directly benefitted from the expansion of pharmacy services, including expanded direct patient care services, medication adjustment and monitoring through the CPAs, and clinical medication profile reviews for optimal chronic disease management.

Providers are regularly asked for feedback on the intern program and have not described any concerns with the transition of responsibilities from pharmacist to intern. Several providers have expressed appreciation for having the interns available on-site to answer insurance-related questions when the pharmacist is not at the clinic. Periodically, a patient will tell the provider during their office visit that they cannot afford the medication, or the insurance requires some action before approval. The interns have served as a valuable resource and are able to smoothly rectify the situation for the patient and the provider, deferring to the pharmacist when necessary.

Clinic—Implementation of the pharmacy internship program assisted in developing cost-effective pharmacy strategies for both the patient and the clinic. Prior to the fellowship program, other staff members (e.g., medical assistants, staff nurses, or support staff) would need to complete patient paperwork. A 2011 article estimated US physician and administrator costs related to time spent interacting with payers to be almost $83,000 annually [13]. The estimated cost of hiring the interns to manage these interactions with payers and other administrative responsibilities is approximately $10,800 annually, more than a seven-fold cost differential. Reduction in the burden of these administrative processes for providers, clinic staff and pharmacy fellows has increased efficiency in other areas and provides more time to deliver optimized patient-centered care.

Student pharmacist learners—Prior to the pharmacy internship program, APPE students at the clinics spent considerable time on PAs and MAP applications, resulting in few direct patient care activities. With the pharmacy internship program in place, APPE students engage in more hands-on patient interaction experiences, such as independently leading anticoagulation management visits, organizing medication and disease consult appointments, providing clinical recommendations to nurse practitioners through chart review, conducting in-service education to the interprofessional staff, and leading educational sessions with patients. APPE students also have the opportunity to participate in scholarly activities with the fellows and the institutions.

## 5. Challenges in Implementing the Pharmacy Internship Program

There were challenges in the implementation of the pharmacy internship program. First, there was an initial learning curve for the interns in identifying the best ways to contact insurance companies and communicating the information to successfully complete a PA or MAP application. This led to some trial and error in the beginning and resulted in some general delays with completing the daily assigned tasks. To reduce the transitional burden, one intern is recruited annually upon completion of their first professional year in the pharmacy program to fill the place of the intern advancing to the fourth year of the curriculum, while the other intern remains in place, providing stability and consistency during each period of transition. The “senior” intern serves as a resource and assists in orienting and training the “junior” intern. Further, semi-structured interviews occur at the end of each intern’s time in the role to identify areas of improvement or difficulties that can be addressed by the fellows or the organization. Second, the position was originally approved for 12 h a week, which was often evenly split by the interns. This allowed for coverage three to four days a week, however, the clinics are open up to 12 h a day. As such, there was only a brief amount of time for the interns to complete paperwork for all four clinics, and the volume of patients cared for by the providers outpaced what the interns could provide. Requests would sometimes take several days to be processed, especially during holidays and weekends. The program expanded its hour limit to 16, which offset some of these delays and helped ensure timely processing of all responsibilities. Third, as part of their education, the pharmacy interns had academic requirements that periodically kept them from being able to work at the clinic, such as final exams or IPPEs. This challenge was managed by using a flexible schedule approach, requiring the interns to identify when their external responsibilities would be heaviest. The interns were able to adequately balance their academic and co-curricular needs through open communication with the pharmacist fellow, and no significant delays in service occurred.

## 6. Conclusions

This pharmacy internship program in an outpatient primary care organization is a unique learning and employment opportunity for student pharmacists. It provides them with a valuable experience while simultaneously benefitting the clinics, providers, and patients. From the intern perspective, working with the medically underserved was considered the most beneficial aspect of the program. Further, the interns utilized their acquired skills and experiences from the internship program during their APPEs. This experience also encouraged them to explore opportunities to work with underserved patient populations. The program demonstrates improvement in provider efficiency by spending less time on administrative paperwork and creates opportunities for the provision of direct patient care. Additional benefits are seen for the organization and student learners on required learning rotations. Primary care facilities may benefit from implementation and expansion of the pharmacy intern role to other basic clinical pharmacy needs.

## Figures and Tables

**Table 1:** Pharmacy intern completed tasks.

October 2017–October 2018	
Assigned Tasks	Volume Completed
PAs	1582
MAP applications	208
Appeals to PA rejections	21
Contraceptive device procurement	3
Inventory	4
Other tasks completed	252
Total tasks completed	2070

MAP = medication assistance program; PA = prior authorization.
